# Uses, Limitations, and Validity of a Registry of Congenital Anomalies in Iran: A Critical Review

**DOI:** 10.1155/2017/6972617

**Published:** 2017-07-11

**Authors:** David H. Stone, Saeed Dastgiri, Mohammad Heidarzadeh, Hossein M. Abdollahi, Shahin Imani, Mohammad H. K. Maher

**Affiliations:** ^1^PEACH Unit, Yorkhill Hospital, University of Glasgow, Glasgow, UK; ^2^Tabriz Health Services Management Research Centre, Tabriz University of Medical Sciences, Tabriz, Iran; ^3^Ministry of Health, Tehran, Iran; ^4^Pediatric Health Research Centre, Tabriz University of Medical Sciences, Tabriz, Iran

## Abstract

**Background and Aims:**

Preventive strategies of congenital anomalies are basically relying on the systematic ongoing collection and analysis of data and timely dissemination of information. The aim of this paper is to briefly report a critical review of a surveillance system of congenital anomalies in a developing country, by describing the challenges and experience of the registry since it began.

**Methods:**

Tabriz Registry of Congenital Anomalies (TRoCA) was mainly set up based on the guidelines provided by the International Clearinghouse for Birth Defects Surveillance and Research (ICBDSR) for data collection, coding, process, analysis, use, and evaluation of the system.

**Findings:**

TRoCA has successfully achieved its main objective as a pilot model for setting up a nationwide registry of congenital anomalies in the country. The programme has too succeeded in relation to its regional objectives: epidemiological rates and data have been produced consistently for etiological investigations, methodological studies, service provision, and preventive measures for selected anomalies.

**Conclusions:**

Our successful experience, as a small registry in a developing country, might be of interest and useful to practitioners, policymakers of birth defects control programmes, and mainly those willing to set up a monitoring system of congenital anomalies in similar areas.

## 1. Introduction

Birth defects are making a proportionally major contribution to perinatal mortality, childhood morbidity, and disability in many countries. Occurrence of congenital disorders varies between different countries ranging from 2 to 10 percent of births [1, 2].

The prevention of congenital anomalies requires prior knowledge of the aetiology and causal factors involved. Although aetiology is still largely unknown, preventive methods are now available for about 60 percent of congenital abnormalities [3, 4]. Preventive strategies, on the other hand, are basically relying on the surveillance, systematic ongoing collection and analysis of data, and timely dissemination of information. To assure the quality of these measures, critical review of the procedures for evaluation purposes has previously been introduced for monitoring systems [5, 6].

The aim of this paper is to briefly report a critical review of a monitoring system of congenital anomalies in Iran, by describing the challenges and experience of the registry since it began.

## 2. Methods

### 2.1. Tabriz Registry of Congenital Anomalies

In 2000, a project was carried out in the Tabriz city of Iran to investigate the epidemiology of congenital anomalies. The aim of the study was to provide baseline information to set up a regional registry of birth defects for the first time in the country. This programme was then called Tabriz Registry of Congenital Anomalies (TRoCA) [7]. Our programme was also accepted in the International Clearinghouse for Birth Defects Surveillance and Research (ICBDSR), and European network of registries for congenital anomalies (EUROCAT) as a member of countries having an established registry for birth defects [1, 2].

Some of the registry systems of the ICBDSR and EUROCAT members were studied in terms of data collection, process, analysis, use, and evaluation of the system to determine the requirements for setting up a local registry in Iran. The minimum requirements for the registry were also determined.

TRoCA is located in Tabriz city run under the Tabriz University of Medical Sciences. Tabriz is one of the three major cities in Iran, located in the northwest region. Tabriz University of Medical Sciences is one of the five top universities in the country providing medical and health services for the population in the northwest of Iran. TRoCA has been financially supported by local and national funds.

### 2.2. Objectives

The principal aims of TRoCA programme are to establish a monitoring system of congenital anomalies in the region and to implement control and preventive tasks in the area. It was primarily intended to use TRoCA framework as a pilot model for setting up a surveillance of birth defects in the whole country. Purposes of TRoCA are to

- register the occurrence of selected birth defects in the region,
- prepare epidemiological indexes to indicate the magnitude and trends over time,
- monitor emerging or unusually high occurrences of congenital anomalies,
- make valid data available to policymakers,
- plan and implement preventive and control strategies to prevent selected anomalies,
- evaluate prevention and control strategies.

### 2.3. The Registration Process and Methodology

Prenatal care is routinely provided for every pregnant woman on a regular basis (up to eight times) with 1–3 diagnostic sonographies during pregnancy. If needed, further diagnostic procedures (i.e., genetic tests for congenital anomalies, etc.) are performed. Termination of pregnancies is permitted for a few selected anomalies. TRoCA reports termination rate for major malformations only. After birth, all children in three hospitals involved in the programme are normally examined by a gynaecologist, obstetrician, neonatologist, or pediatrician at birth. They are followed up until hospital discharge for general health status, maturity, and congenital anomalies. The TRoCA programme covers about 15,000 births (annual average) in the area with about 300–400 newborns with one of the anomalies in this population. Background information and basic characteristic data are gathered for all births in TRoCA region. Some additional information (i.e., family history, parity, parental age, residence, education, maternal illness, gestational length, birth weight, and type of birth) is also available for infants with anomaly and mothers. Karyotype and autopsy are not routinely performed unless it is requested as a necessity after full clinical investigation.

We use a “passive” method of data collection. The responsible persons (as registrar) for data documentation are midwives. A medical coder has been assigned in this programme to code/classify the defects. The end users defined the congenital anomalies for the purposes of this programme based on the standard coding system of the International Classification of Diseases (ICD) under one of the following main headings according to the primary diagnosis of anomaly:

- Nervous system anomalies;
- Genitourinary tract and kidney;
- Anomalies of limb;
- Chromosomal anomalies;
- Cleft lip with/without palate;
- Congenital heart disease;
- Musculoskeletal and connective tissue anomalies;
- Digestive system anomalies;
- Eye and ear anomalies;
- Other anomalies.

Total prevalence is calculated by dividing the numerator (registered cases of congenital anomalies in the TRoCA region) by the relevant denominator (total live and stillbirths in the TRoCA region) for the same period of time. An infant/fetus with more than one anomaly is counted once only in the numerator. The main criteria of inclusion of fetal deaths or stillbirths in data analysis are pathologic confirmation of the defect provided by the hospitals. Time trend analysis, relative frequencies, and confidence intervals are also calculated for some statistical purposes. For more details of the methodology, TRoCA publications may be searched.

### 2.4. Ethics

TRoCA activities have been approved by the Ethics Committee of the Tabriz University of Medical Sciences. Confidentiality and privacy of identity-related information are strictly considered in every part of the data gathering, handling, processing, registration, access, and reports.

## 3. Findings

### 3.1. Uses of TRoCA in Relation to Its Objectives

#### 3.1.1. Detection of Epidemics

To date, generally, our data shows no epidemic of any of kinds of birth defects in the area. However, an unusual increase occurred for the total prevalence of congenital anomalies in 2002 in the registry region. We then identified that this happened due to an improvement in case ascertainment.

#### 3.1.2. Time Trends

**Figure 1:**
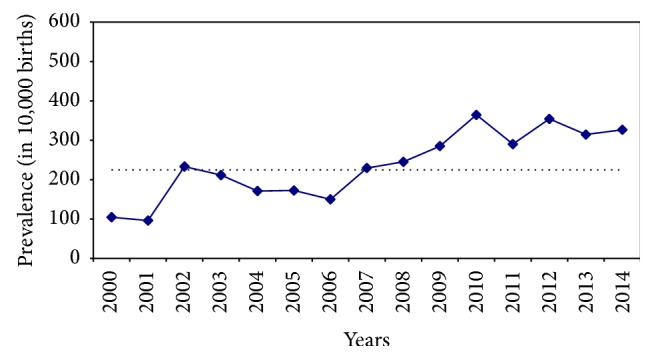
Time trend of prevalence of congenital anomalies (Tabriz, Iran) (dotted line shows the total prevalence in average).

**Figure 2:**
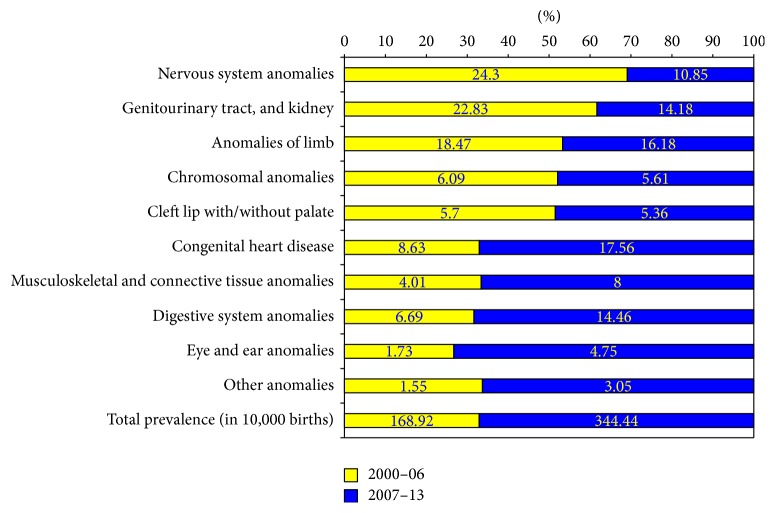
Comparison of the defects proportion (%) between 2000–06 and 2007–13 (the last bar shows the total prevalence in 10,000 births).

As seen in Figure 1, there is a steady increase in the occurrence of congenital anomalies in the region over time. Total prevalence of anomalies is more than tripled ranging from 104.6 (per 10,000 births) in 2000 to 326.5 (per 10,000 births) in 2014. During this period of time, early records show that nervous system and genitourinary tract anomalies were the most frequent defects while later data indicated that heart and limb defects are the most common ones (Figure 2).

#### 3.1.3. Estimates of Prevalence

**Figure 3:**
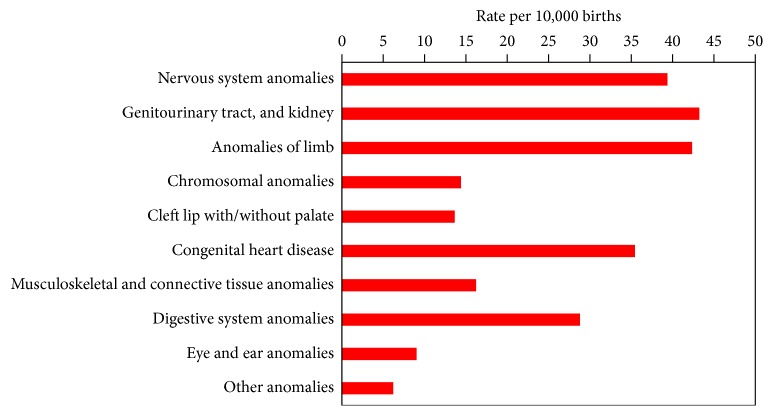
Prevalence of congenital anomalies (Tabriz, Iran).

A total of 261 024 births were registered in the region over the study period including 258 153 (98.9%) live births and 2871 (1.1%) stillbirths. During this period, 5870 cases with a primary diagnosis of congenital anomaly were ascertained, representing an overall prevalence rate of 224.9 per 10,000 births. Genitourinary tract and kidney anomalies, limb defects, anomalies of nervous system, and congenital heart diseases accounted proportionally for more than 65 percent of anomalies in the region (Figure 3).

#### 3.1.4. Geographical Variations

Table 1 shows the prevalence (in 10,000 births) of selected congenital anomalies in 13 ICBDSR registries across the globe published in 2014 for the data between 2007 and 2011 [2]. The rate of total limb reduction defects in TRoCA is almost nine times, in average, higher than that of other regions. Tabriz displays almost similar rates for other groups of birth defects compared to other registries, although the rate of anencephaly, hydrocephaly, and cleft palate is still high in the region while spina bifida shows a low rate.

#### 3.1.5. Special Studies

Data provided by TRoCA have resulted in a study to estimate the missing frequency of congenital cardiac anomalies at the time of delivery and birth in the region. Accordingly, 59.1% of children with congenital heart diseases were not identified at birth [8].

We found that the accuracy of family physicians in case detection and diagnosis of congenital anomalies in rural areas is more than 98% [9]. We also investigated the occurrence rate (33 percent) of termination in pregnancies with congenital anomalies [10], and association of folic acid consumption and birth defects [11]. More other studies have been carried out using our registry data. For details, TRoCA publications need to be searched.

#### 3.1.6. Response to the Needs and Services

In addition to the registration of birth defects, TRoCA has extended its activities to implement control and preventive services for genetic disorders and congenital anomalies in the region. This includes genetic services to families who have a history of an anomaly/disorder in the family, and preconceptional programmes for young couples. This new programme called Tabriz Foundation for Public Health Genetics (TFPHG) was launched in 2013 [12].

#### 3.1.7. Etiological Studies

General information is routinely collected for every neonate. Some exposure information is also available of mothers of all malformed infants. Other women giving births in the TRoCA maternity and children hospitals with normal newborns routinely complete the similar data form. They might be considered as matched control group. Using these data of control group plus routine statistics from general population [13], testing of etiological hypotheses and investigation of the role of some exposures are virtually possible in TRoCA registry.

### 3.2. Limitations of TRoCA

#### 3.2.1. Epidemiological Pitfalls

TRoCA is always able to describe the distribution and occurrence of congenital anomalies in its defined population (by time, place, and other influencing factors). However, epidemiological reliability and representativeness of the rates captured by the programme have not been fully investigated yet. The timeliness of the information provided by TRoCA is also still a matter of epidemiological pitfall where the programme is able to release primary information on the occurrence of congenital anomalies at least one year after the data collection.

As indicated before, TRoCA programme monitors about 15,000 births (annual average) in the area with about 300–400 cases with one of the defects in the newborns. The very small frequency and the rare nature of some groups of anomalies influence the epidemiological power of the rates and occurrence patterns of various types of congenital anomalies provided by the programme.

#### 3.2.2. Delayed Ascertainment

Multiple sources of case ascertainment result in a true pattern of birth defects in the population. The data of TRoCA comes mainly from two maternity hospitals plus a referral medical centre for sick children in the region. Some anomalies are routinely not diagnosed until some times after birth. For this reason, ascertainment of those defects is inevitably delayed. Nearly 60% of children born with congenital heart disease, for example, were not ascertained by TRoCA at birth. They were then identified as having heart defects in children hospital during the next 12 months after birth [8]. The delayed ascertainment, therefore, appears to be an inevitable part of the registry.

#### 3.2.3. Variable Validity of Data over Time

At birth, nervous system anomalies were ranked first in the TRoCA earlier years' data while the first rank belongs to congenital heart defects in recent years' records (Figure 2). The reason is that the improvement of the diagnosis and identification of congenital heart defects over time have resulted in a complete ascertainment of these groups of anomalies in the registry. This variation in the ascertainment over time appears to be an inevitable limitation of every registry including TRoCA.

## 4. Discussion

### 4.1. Has the TRoCA Succeeded?

Tabriz Registry of Congenital Anomalies started its main activities in 2000. A nationwide registry of congenital anomalies in Iran was then established in 2012 based on the data, framework, and baseline structure provided by TRoCA (as a pilot programme). It is therefore believed that TRoCA has successfully achieved its main objective as a pilot model for the whole country.

TRoCA has too seemingly succeeded in relation to its regional objectives: annual prevalence rates have been produced consistently over years [7], geographical comparisons have been made possible by linking the TRoCA with international programmes [1, 2], and TRoCA has provided data for some specific investigations [10, 11], service provision for selected anomalies [8], and created a validated tool for family practitioners for case detection and diagnosis of birth defects [9]. In response to the health care needs of high risk population, TRoCA is implementing control and preventive services for some genetic disorders and anomalies in the region [12].

### 4.2. Main Challenges

TRoCA recorded a low rate for spina bifida, high rates for anencephaly, hydrocephaly, and cleft palate without cleft lip, and a very high rate of limb reduction defects in the region. While TRoCA examines and reports the annual rates of congenital anomalies consistently, we do not know for sure whether any change in these rates is due to a true existence/absence of epidemics in the region, due to the technical failure of our monitoring, or due to aetiologic and environmental teratogens. We do not have any parallel surveillance for environmental teratogens. The reasons behind the high/low rates of some groups of anomalies are still unclear. There need therefore for further investigations for every selected anomaly with unusual rate of occurrence in the area.

As a programme in a developing country, the condition of antenatal screening procedures, detection and ascertainment methods of defects, variable validity of the data, low coverage and small denominator population (due to the limited sources of funding), small number of cases (due to the small population covered), low power of the programme to find rare defects, and no full follow-up plan for anomalies after hospital discharge are still among the main limitations of the registry. The lack of epidemiological power and representativeness should therefore be carefully considered in examining the rates and patterns of various types of congenital anomalies, and when investigating for possible local causes and influencing factors of birth defects in the area.

For evaluation purposes, we assess the strengths and weaknesses of the TRoCA programme based on the major evaluation components of a standard monitoring system (i.e., simplicity, flexibility, acceptability, reliability, utility, sustainability, timeliness, sensitivity, and representativeness).

TRoCA has, so far, relied on staff for whom this programme was not their core duties. Allocating fully funded full time individuals for TRoCA may help to solve some parts of the above problems in its future activities.

It is concluded that as a small registry of congenital anomalies in a developing country, although TRoCA is still facing some challenges and problems, we believe that it has been successful in achieving its main objectives. Our experiences might be of interest and useful to practitioners, policymakers of birth defects control programmes, and mainly those willing to set up a monitoring system of congenital anomalies.

## Figures and Tables

**Table 1:** Prevalence (in 10,000 births) of selected congenital anomalies in 13 ICBDSR registries^(1), (6), (7)^.

Selected congenital anomalies	Australia: WARDA^(2)^	Canada	France: Paris	Germany: Saxony-Anhalt	India^(3)^	Iran: Tabriz	Italy: Tuscany	Japan	Malta^(3)^	Saudi Arabia^(3)^	South America^(4)^	Ukraine	USA: Texas
Anencephaly	5.5	1.2	4.9	2.0	11.8	**10.7**	1.5	0.7	1.5	10.1	5.8	7.0	2.7
Spina bifida	5.7	3.2	5.2	5.2	11.8	**1.4**	3.9	5.6	8.4	2.0	8.5	10.4	3.9
Cleft palate without cleft lip	10.2	6.4	6.4	5.9	1.7	**8.4**	3.3	5.1	14.3	2.0	4.5	7.2	5.9
Encephalocele	2.1	0.7	2.2	1.4	2.5	**1.0**	1.0	0.5	1.9	2.0	3.3	1.8	1.0
Microcephaly	3.8	3.7	2.4	11.7	1.4	**4.5**	0.6	1.6	4.5	12.1	4.8	5.9	12.9
Hydrocephaly	7.7	4.7	14.4	4.6	8.9	**13.8**	3.4	7.8	1.9	12.1	17.2	5.3	7.2
Cleft lip with or without cleft palate	10.8	9.4	8.2	12.1	4.6	**11.9**	5.2	21.7	10.9	14.1	11.7	7.8	10.4
Undescended testis	39.5	34.4	NR^(5)^	4.7	0.9	**9.8**	6.6	NR	NR	NR	9.8	31.6	13.7
Hypospadias	34.6	27.5	17.3	6.9	1.8	**19.8**	13.9	5.2	22.7	14.1	9.2	2.8	16.6
Limb reduction defects	7.0	3.3	6.1	7.7	4.2	**51.0**	4.2	3.8	7.4	4.0	7.6	4.8	6.0
Polydactyly	12.1	13.2	1.9	5.5	3.2	**8.0**	1.1	6.7	17.3	10.1	3.1	4.1	4.0
Tetralogy of fallot	2.9	3.4	4.1	2.8	0.4	**1.4**	2.3	7.0	2.9	6.0	1.6	2.7	3.9
Coarctation of aorta	4.3	4.5	3.8	5.4	0.1	**2.9**	2.3	6.7	3.5	2.0	0.5	1.7	5.2
Hypoplastic left heart syndrome	2.2	2.4	3.1	2.7	1.1	**3.7**	2.6	4.5	5.9	4.0	0.9	2.0	2.2

*Notes.* ^(1)^Data based on the annual report (2014) published by the International Clearinghouse for Birth Defects, Surveillance and Research (ICBDSR); ^(2)^Western Australian Register of Developmental Anomalies; ^(3)^data based on the annual report (2012) published by the International Clearinghouse for Birth Defects, Surveillance and Research (ICBDSR); ^(4)^Latin American Collaborative Study of Congenital Malformations; ^(5)^not Reported; ^(6)^for the details of the populations covered in each registry, please refer to the ICBDSR website at: <http://www.icbdsr.org>; ^(7)^registries in this table are listed in alphabetic order.

## References

[1] European Concerted Action on Congenital Anomalies and Twins (EUROCAT), 2016, http://www.eurocat-network.eu

[2] International Clearinghouse for Birth Defects Surveillance and Research (ICBDSR), 2016, http://www.icbdsr.org

[3] Czeizel A. E. (1993). Prevention of congenital abnormalities by periconceptional multivitamin supplementation. *British Medical Journal*.

[4] Czeizel A. E., Intody Z., Modell B. (1993). What proportion of congenital abnormalities can be prevented?. *British Medical Journal*.

[5] Lee M. L., Teutsch S. M., Thacker S. B., Louis M. E. St. (2010). *Principles Practice of Public Health Surveillance*.

[6] Stone D. H. (1989). The glasgow register of congenital anomalies 1972-88: a critical review. *Journal of Inherited Metabolic Disease*.

[7] Tabriz Registry of Congenital Anomalies (TRoCA), 2016, http://troca.tbzmed.ac.ir

[8] Dastgiri S., Taghizadeh M., Heidarzadeh M. (2011). Early diagnosis and screening of congenital cardiac anomalies. *Cardiology in the Young*.

[9] Mashhadi Abdolahi H., Kargar Maher M. H., Karamouz M., Khosroshahi H., Dastgiri S. (2014). How Accurate Is Diagnosis of Congenital Anomalies Made by Family Physicians?. *Health Promotion Perspectives*.

[10] Samadirad B., Khamnian Z., Hosseini M. B., Dastgiri S. (2012). Congenital anomalies and termination of pregnancy in Iran. *Journal of Pregnancy*.

[11] Hosseini M. B., Khamnian Z., Dastgiri S., Samadi Raad B., Ravanshad Y. (2011). Folic acid and birth defects: a case study (Iran). *Journal of Pregnancy*.

[12] http://www.tfphg.com.

[13] Statistics Administration Organization, https://amar.org.ir

